# Circulating proteins as predictive and prognostic biomarkers in breast cancer

**DOI:** 10.1186/s12014-022-09362-0

**Published:** 2022-07-11

**Authors:** Hugo Veyssière, Yannick Bidet, Frederique Penault-Llorca, Nina Radosevic-Robin, Xavier Durando

**Affiliations:** 1 Université Clermont Auvergne, INSERM, UMR 1240 « Imagerie Moléculaire Et Stratégies Théranostiques », Centre Jean Perrin, 58 Rue Montalembert, 63011 Clermont-Ferrand, France; 2 Division de Recherche Clinique, Délégation Recherche Clinique & Innovation, Centre Jean Perrin, 58 Rue Montalembert, 63011 Clermont-Ferrand, France; 3 Centre d’Investigation Clinique, UMR501, 63001 Clermont-Ferrand, France; 4 Département d’Oncologie Médicale, Centre Jean Perrin, 58 Rue Montalembert, 63011 Clermont-Ferrand, France; 5 Département d’Oncogénétique, Laboratoire d’Oncologie Moléculaire, Centre Jean Perrin, 58 Rue Montalembert, 63011 Clermont-Ferrand, France; 6 Département d’Anatomie et de Cytologie Pathologiques, Centre Jean Perrin, 58 Rue Montalembert, 63011 Clermont-Ferrand, France

**Keywords:** Breast cancer, Liquid biopsy, Serum proteins, Plasma proteins, Prognostic, Predictive, Biomarker

## Abstract

Breast cancer (BC) is the most common cancer and among the leading causes of cancer death in women. It is a heterogeneous group of tumours with numerous morphological and molecular subtypes, making predictions of disease evolution and patient outcomes difficult. Therefore, biomarkers are needed to help clinicians choose the best treatment for each patient. For the last years, studies have increasingly focused on biomarkers obtainable by liquid biopsy. Circulating proteins (from serum or plasma) can be used for inexpensive and minimally invasive determination of disease risk, early diagnosis, treatment adjusting, prognostication and disease progression monitoring. We provide here a review of the main published studies on serum proteins in breast cancer and elaborate on the potential of circulating proteins to be predictive and/or prognostic biomarkers in breast cancer.

## Background

Breast cancer (BC) is the most common cancer and among the main causes of cancer death in women [1]. It is a heterogeneous disease, consisting of a number of morphological and molecular subtypes. Molecular analyses allow dividing BC into three groups: luminal BC (expressing estrogen receptor /ER + / or progesterone receptor /PR + /), HER2-enriched BC (overexpressing human epidermal growth factor receptor 2 and/or having the HER2 gene amplified, without expression of ER or PR) and triple negative breast cancer (TNBC, ER, PR − , and HER2 −) [2, 3]. ER + BC are divided into luminal A and luminal B tumours. Luminal A is the most common subtype (40% of all BC), and is associated with low expression of proliferation-related genes. Among all BC, this is the subtype with the best prognosis and the lowest risk of recurrence [4]. Luminal B subtype represents almost 20% of all BC and is associated with high expression of proliferation-related genes and high risk of recurrence [4]. Hormone therapy remains the optimal treatment for luminal BC. In HER2-enriched BC (10–15% of all BC) the presence of amplified *ERBB2*, the HER2 gene, allows the use of targeted anti-HER2 therapy which has significantly improved the prognosis of HER2 + BC in recent years. Finally, TNBC, that accounts for 10 to 20% of all BC, is the most heterogeneous and aggressive subtype. Due to ER, PR, and HER2 negativity, TNBC is treated by chemotherapy, to which is more sensitive than other types of BC, however still has a poor prognosis due to high rate of metastatic recurrence. Most TNBC recurrences occur during the five years after the diagnosis and the median survival of patients in the metastatic phase is inferior than 15 months [5, 6]. TNBC heterogeneity and aggressiveness have led during the last decades to massive efforts to find reliable prognostic and predictive biomarkers in this subtype of BC. Therefore, a large majority of recent studies on BC biomarkers focus on TNBC.

Biological and clinical behaviour of breast cancer clearly varies from one patient to another, making predictions of disease evolution and patient outcomes difficult. Therefore, biomarkers are needed to help clinicians choose the best treatment for each patient. For years, breast cancer treatment has been led by tissue-based biomarkers, like estrogen receptor, progesterone receptor and HER2 status [7]. However, many studies on breast cancer have also demonstrated that almost all cancers release their components into the circulation [8–10]. Thus, blood can be used to analyze biomolecules originating from tumours.

Consequently, “liquid biopsies” are more and more frequently performed. They are less invasive than tissue biopsies and appear as an alternative for discovering new biomarkers. Numerous studies focused on potential biomarkers in blood such as circulating tumour cells, blood cells, circulating genetic material (circulating tumoral DNA, miRNA or exosomes) [11–13] or proteins. Circulating proteins, blood components or secreted by tumours, are involved in various biological functions and, as such, are an important source of cancer biomarkers. Blood proteome contains a set of tissue proteome [14]. Thus, plasma proteins and/or plasma protein level changes provide information about the physical condition and health status of patients and can be used to track disease progression [15]. Cancer cells acquire several capabilities for facilitating progression and metastasis such as manipulation of the immune response, growth stimulation, or induction of angiogenesis and invasion [16]. These acquired abilities can be promoted or reflected, among other elements, by circulating proteins.

Circulating proteins, from tumours or produced by the immune system, play an important role in the development and progression of breast cancers [17]. Proteins can act as the primary bioeffectors of metastasis. The cancer secretome consists of all proteins secreted or shed by cancer cells into the extracellular compartment or bodily fluids, and can promote cancer progression [18, 19]. The cancer-secreted proteins (enzymes, cytokines, and growth factors) are involved in various biological and physiological processes such as immune response and cell–cell communication. An important part of the cancer secretome can be found in measurable amounts in blood. Those proteins are potential biomarkers, easier to access than the proteins within the tumour tissue. Various teams studied the cancer cell secretome through mass spectrometry or antibody array, two major techniques of proteome analysis [8].

Secretome analysis is one of the ways to get insight into the tumour microenvironment. The tumour microenvironment consists of the extracellular matrix, immune and non-immune benign cells, blood vessels and a plethora of secreted molecules, mostly proteins. This microenvironment and the tumour interact with each other, and exchanges between them can induce the stimulation and/or activation of signaling pathways contributing to tumour progression, including the acquisition of a malignant phenotype [20]. The cancer secretome stimulates tumour evolution by promoting invasion and metastasis [18]. Functional analyses in 32 cancer types demonstrated that cancer cells secrete proteins which promote proliferation and invasion [21].

The promotion of metastasis by the cancer secretome is one of the key features of instigation, a process by which the primary tumour stimulates dormant cancer cells at distance or prepares a metastatic niche to accept the circulating metastatic cells [22, 23]. The existence of instigation is the basis for the search of metastatic recurrence biomarkers among the molecules circulating in blood [24, 25]. McAllister et al. demonstrated that dormant cancer cells can be stimulated by cytokines secreted by the bone marrow-derived mesenchymal cells to develop into the metastatic collections [21]. Today, no circulating protein is considered to be the specific reactivator of dormant cells. However, high concentrations of some proteins involved in inflammation, such as interleukin-6 (IL-6) and interleukin-8 (IL-8), or in angiogenesis, such as angiopoietin-like proteins, have been shown to be associated with a high risk of metastatic progression of breast cancer [27–29].

Today, non-invasive methods for sampling materials for biomarkers are being intensely developed. Blood protein assessment is a straightforward method that can be performed routinely and frequently. The quantification of circulating proteins has been a part of clinical analysis for a long time. In addition, a wide range of techniques for protein assessment, including enzyme-linked immunosorbent assay (ELISA), mass spectrometry (MS), antibody array or aptamer-based proteomics, has been developed during the last decades. Recently, the capacity and the specificity of these assays have been significantly increased through the development of technologies that allow the assay of hundreds or even thousands of proteins, including the low-abundant ones. Thus, targeted blood proteomics appears as a promising way to discover new biomarkers, thanks to the use of high-throughput techniques.

**Fig. 1:**
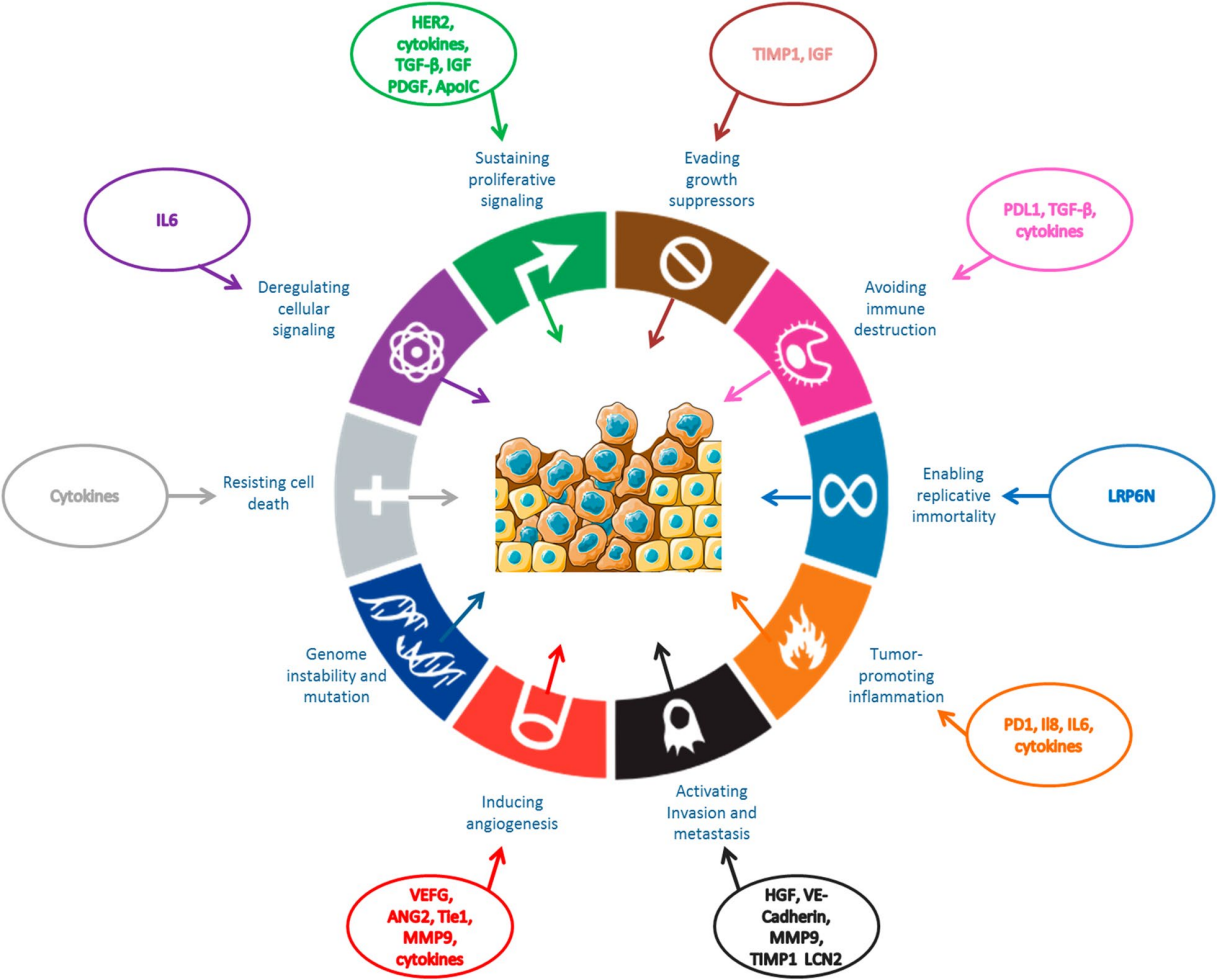
Proteins detailed in this review cover most of the hallmarks of cancer established by Hanahan and Weinberg. This adapted figure from Hanahan and Weinberg’s review [16] shows the impact of circulating blood proteins involved in breast cancer on different cancer hallmarks

In this review, we summarize about the tumour-produced circulating proteins (Table1) and circulating proteins produced by the immune system (Table 2) that have a potential to serve as predictive or prognostic biomarkers in breast cancers. Proteins detailed in this review are involved in most of the hallmarks of cancer established by Hanahan and Weinberg (Fig. 1) [10].

**Table 1:** Studies of interest presenting tumoral circulating proteins in plasma and serum of patients with breast cancer

Protein	Function	Studied population	Number of patients	Clinical association with elevated blood levels	Refs
VEGF	Angiogenesis factor	BC	44	Poor disease progression and late clinical stages	[96]
		Metastatic BC	253	Poor clinical outcome and poor overall survival	[75]
		TNBC	21	At baseline, associated with a good overall survival (10.2 months vs 4.2 months in the low levels group)	[99]
		TNBC, non-TNBC and healthy participants	43 (TNBC) 53 (non-TNBC) 20 (healthy participants)	Worse response to NAC, metastasis and a poorer OS (median OS around 22 months)	[63]
		TNBC	303	Unfavourable outcome (3-year DFS of 53% vs 85% with low serum VEGF levels)	[100]
TGF β	Cytokine controlling proliferation	BC and healthy particpants	44	Advanced stages	[61]
		BC	60	Advanced stages	[62]
		TNBC	43	High incidence of metastasis, relapse, and poor response to NAC	[63]
		TNBC	48	TGF-β-related proteins were associated with TNBC tumour progression and poor outcomes	[64]
		oesophageal adenocarcinoma	NA	TGF-β serum levels in metastatic patients were significantly higher compared to patients with non-metastatic disease	[65]
MMP9	Endopeptidase involved in the degradation of extracellular matrix	BC and benign breast disease	77 (BC) 10 (benign)	Higher in the breast cancer group compared to the benign tumour group	[91]
		Metastatic BC and BC	88 (M +) 160 (M0)	Prognostic factor	[72]
		TNBC	303	High levels correlated with a decrease in pCR rate and a poor response to NAC	[93]
HER2	Involved in the regulation of cell proliferation	BC	118	Elevated preoperative correlated with a worse prognosis	[69]
		HER2 + M + BC	537	Short PFS	[70]
		BC	64	Poor outcomes	[71]
		Metastatic BC and BC	88 (M +) 160 (M0)	Appearance of brain metastasis	[72]
TIMP-1	Matrix metalloproteinases inhibitor	Metastatic BC	253	Poor PFS and OS	[74]
		BC	60	Elevated serum levels associated with lower progression-free and OS rates	[76]
		HER2 + M + BC	472	TIMP-1 levels described as prognostic factor of shorter PFS	[70]
TIE1/2 and Ang-2	Promotes angiogenesis	BC and healthy participants	143 (BC) 100 (healthy patients)	Ang-2 was higher in BC than in the healthy group and was associated with a worse OS and metastasis	[106]
		BC and benign breast disease	127 (BC) 38 (benign breast disease)	Ang-2 was higher in BC than in the benign breast disease group	[107]
		M + BC	181	High Ang-2 levels at baseline associated with poor PFS. Both Ang-2 and serum Tie2 appeared as prognostic factors of poor OS	[108]
		M + BC	58	Good prognostic value, short median OS (around 20 months) and short median PFS	[109]
HP	Captures haemoglobin during haemolysis and inhibits oxidative activity	BC	6606	Poor clinical outcomes	[113]
		TNBC and healthy participants	41 (TNBC) 10 (healthy patients)	Poor prognosis and low survival rate	[114]
		TNBC and non-TNBC	30 (TNBC) 30 (non-TNBC)	High expression in TNBC group compared to non-TNBC group	[115]
CA15-3 and CEA	Prognostic markers for BC	TNBC	247	Elevated CEA and CA15-3 levels associated with short OS and DFS rates	[118]
		TNBC	604	High risk of death	[119]
VE-cadherin	Plays a crucial role in endothelial adherens junction assembly and maintenance	BC	48	Predictive factor for recurrence	[83]
		Hormone-resistant M + BC	141	Prognostic factor for both PFS and OS	[84]
HGF	Involved in morphogenesis, cell and tissue survival, and cellular growth	BC	134	Appearance of metastases	[78]
		BC	34	Poor prognosis and a high risk of progression	[79]
		BC	121	Metastasis	[80]
IGF-I and PDGF	Regulator of growth, survival, migration and invasion	TNBC, non-TNBC and healthy participants	43 (TNBC) 53 (non-TNBC) 20 (healthy participants)	Metastasis and recurrence	[63]
		BC	110	Associated with high serum PDGF levels, IGF-I may increase this risk of recurrence	[110]
LRP6N	Co-receptor for Wnt signal induction	BC	295	Diagnostic marker for the early detection of breast cancer metastasis	[77]
PD1/PDL1	Immune checkpoints	TNBC	66	Predictive factors of treatment response	[132]
		HER2 + M + BC	387	High serum PDL1 level before treatment strongly linked to longer OS in the lapatinib group compared to the trastuzumab group	[133]
		M + BC	208	Short PFS of metastatic breast cancer and a poor prognosis	[134]

Ang2: Angiopoietin 2, ApoC-I: Apolipoprotein I-C, BC: Breast cancer, CA15-3: Cancer antigen 15–3, CEA: Carcinoembryonic antigen, ELISA: Enzyme-linked immunosorbent assay, HER2: Human epidermal growth factor receptor 2, HGF: Hepatocyte growth factor, HP: Haptoglobin, IGF: Insulin-like growth factor, LRP6N: LRP6 ectodomain, MMP9: Matrix metalloproteinase 9, M + : metastatic, NAC: Neoadjuvant chemotherapy, OS: Overall survival, pCR: pathological complete response, PDGF: Platelet-derived growth factor, PD1: Programmed cell death protein 1, PDL1: Programme death ligand 1, PFS: Progression free survival, TGF β: Transforming growth factor β, Tie-1/2: Tyrosine kinase with immunoglobulin and epidermal growth factor-homology domains ½, TIMP-1: Tissue inhibitor of metalloproteinase 1, TNBC: Triple negative breast cancer, VEGF: Vascular endothelial growth factor

**Table 2:** Studies of interest presenting circulating proteins produced by the immune system in plasma and serum of patients with breast cancer

Protein	Function	Studied population	Number of patients	Clinical association with elevated blood levels	Refs
IL-6 and Il8		Hormone resistant BC	87	Prognostic factor of survival. Associated with a poor survival	[98]
		BC	110	High serum IL-6 and IL-8 associated with advanced clinical disease stages and lymph node metastasis	[137]
		TNBC	110	High risk of recurrence and metastasis, and poor survival rate	[28]
		M + BC	181	Poor progression-free survival and poor OS	[108]
		HER2 +	249	High risk of distant recurrence	[138]
IFN-γ		ER + BC	72	Favourable disease outcome	[139]
InterleukinsMIP-1α and β		BC	11	High levels of IL-8, MIP-1 alpha, and MIP-1 beta in BC patients	[52]
LCN2	Involved in inflammatory response, and cancer growth	BC and healthy participants	113 (BC) 30 (healthy patients)	Poor clinical outcome	[142]
		BC	303	Poor disease-free survival	[143]

BC: Breast cancer, IL6 and 8: Interleukins 6 and 8, LCN2: Lipocalin 2, MIP-1: Macrophage Inflammatory Proteins-1 alpha M + : metastatic, OS: Overall survival, TNBC: Triple negative breast cancer

## Methods of circulating protein analysis

### Mass spectrometry

MS technology allows large-scale untargeted proteomic and targeted analysis and molecular profiling of tissues or bodily fluids in a rapid and accurate way [30]. A mass spectrometer is composed of an ion source, a mass analyser and a detector, and mass spectrometry techniques separate peptides/proteins according to their mass-to-charge ratio. These techniques have seen a rapid development and several types exist. Indeed, several studies have focused on protein profiling using the matrix-assisted laser desorption/ionisation time-of-flight (MALDI-TOF) MS to determine prostate [31, 32], colorectal [33, 34] or breast [35, 36] cancer biomarkers. As an alternative, surface-enhanced laser desorption/ionisation time-of-flight (SELDI-TOF) MS can be performed. For instance, SELDI-TOF–MS technology was used to screen serum-specific proteins in gastric cancer patients and found a potential biomarker (ApoCIII) for early detection [37]. In ovarian cancer, the profile of serum proteins has also been investigated by SELDI-TOF MS and was useful for the discovery of biomarkers (CA125, haptoglobin, transferrin) [38, 39]. As a consequence, this technology has been recognized for a number of years as an effective technique for finding new biomarkers for cancer diagnosis and prognosis [40]. Today, nano-flow liquid chromatography-MS/MS-based (LC–MS/MS) is the system of choice to assess variations in global blood protein levels [41, 42]. For a few years now, data-independent acquisition has been increasingly performed in wide-scale blood MS studies [43]. It allows provides fragmentation data for many analytes in a specific mass range. Using a method based on LC–MS/MS detection and data-independent acquisition MS, Bruderer et *al.* were able to identify and quantify more than 560 proteins in around 1500 plasma samples [44].

### Immunoassays

As mentioned above, ELISA is also a well-established technique for quantifying proteins in blood and bodily fluids. Briefly, ELISA assays rely on the use of a solid-phase enzyme immunoassay to detect a protein in a liquid sample using antibodies [45]. ELISA is the most commonly used method to measure serum/plasma protein concentrations in lung [46] and breast cancer [14]. By using ELISA to assess serum/plasma proteins, many potential markers of lung cancer (p53, NY-ESO-1, Sox-2) and breast cancer such as CA15-3 have been discovered [46, 47]. Nowadays, ELISA multiplex allows for the measure of multiple proteins in the same sample and at the same time, which markedly improves our ability to detect and quantify circulating proteins. More recently, the proximity extension assay (PEA), a technology developed by Olink Proteomics, allows to analyze secreted proteins in blood [48]. PEA is based on the combination of quantitative real-time PCR with multiplex immunoassays and uses a pair of DNA oligonucleotides linked to antibodies against proteins of interest. In recent years, the PEA technology has even become the most widely used for blood biomarker research in various diseases [48].

### Antibody arrays

Finally, antibody arrays represent another relatively high-throughput technology that enables profiling of a set of proteins of interest at the same time [49]. They are based on the enzyme-linked immunosorbent assay. In antibody arrays, antibodies are fixed on a solid surface and the antibody/antigen interaction is detected by an immunofluorescent reaction. As with ELISA, they are used to profile proteins from tissue or blood samples. In 2003, Miller et al. were the first to perform an antibody microarray to detect biomarkers in the serum of patients with cancers, namely with prostate cancer [50]. In 2010, a team screened the serum expression profiles of 507 proteins in ovarian cancer patients and healthy individuals in an antibody array-based study and found different protein expressions between normal and cancer samples [51]. Recently, the ability of antibody array to screen new biomarkers was validated by ELISA in gastric and breast cancer [52, 53].

### Aptamer based proteomic

In 2010, Gold et al. and Somalogic developed a new proteomic technology based on aptamers which are short single-stranded oligonucleotides that bind with high affinity and specificity to proteins [54, 55]. Since 2010, aptamer based proteomic has been used to determine blood biomarkers in various diseases including ovarian cancer [56], lung cancer [57] or cardiovascular diseases [58].

## Circulating proteins produced by tumour tissue

### Transforming growth factor beta

Transforming growth factor β1 (TGF-β1) is known to play a significant role in the promotion of tumoral progression at the tissue level [59, 60]. Previous studies on serum TGF-β1 in patients with breast cancer showed its correlation with response to treatment and the risk of recurrence [61, 62]. In a study conducted on 44 breast cancer patients, plasma TGF-β1 levels were significantly elevated in advanced-stage (stage IIIB and IV) patients compared with healthy participants, regardless of BC subtype [61]. In six patients undergoing therapy for metastatic disease, the study revealed a decrease in TGF-β1 levels (21 to 51%) for patients who responded to treatment and an increase in TGF-β1 levels (34 to 69%) for BC patients who failed to respond to systemic treatment. Sheen-Chen et al.also demonstrated that breast cancer patients (n = 60) in advanced stages of the disease had high serum TGF-β1 levels [62]. In 2015, one study evaluated the prognostic and predictive values of serum TGF-β1 in triple negative breast cancer (TNBC) (n = 43) [63]. Quantification of TGF-β1 levels before and after chemotherapy has shown a correlation between a high incidence of metastasis, relapse, and poor response to neoadjuvant chemotherapy (NAC) and high serum levels of TGF-β1. For instance, reduced disease-free survival was significantly correlated with high serum levels of TGF-β1. However, TGF-β1 did not appear to differentiate between TNBC and non-TNBC patients. Very recently, quantitative analysis of plasma proteomes was performed to discover proteins predictive of progression and metastases in 48 TNBC patients [64]. A plasma protein-derived TGF-β signature, composed of three proteins (CLIC1, MAPRE1 and SERPINA3), was found. These plasma proteins form a TGF-β-regulated network. High plasma levels of these three TGF-β-related proteins were associated with TNBC tumour progression and poor outcomes. In a larger validation cohort, the cancers of metastatic patients with high levels of CLIC1, MAPRE1 and SERPINA3 recurred within 1.0 to 3.3 years from diagnosis, whereas non-metastatic patients with low levels were recurrence-free during the 3.4 to 4.6 years of follow-up. Interestingly, in patients with oesophageal adenocarcinoma, a cancer transposable to TNBC, Steins et al. revealed that high TGF-β serum levels during neoadjuvant treatment could differentiate the patients with a high risk of metastasis [65]. TGF-β serum levels in metastatic patients were significantly higher compared to patients with non-metastatic disease (45.73 vs 36.25 pg/mL).

### HER2

The extracellular domain (ECD) of the epidermal growth factor receptor 2 (HER2) is the major example of a protein shed by cancer cells It is a transmembrane protein overexpressed in 15% of breast cancers. A proteolytic process releases the HER2 ECD from the receptor and the HER2 ECD is shed from cancer cells into blood circulation [17]. ECD HER2 is considered as a prognosis biomarker in breast cancers that overexpress HER2. Indeed, high HER2 ECD levels correlated positively with parameters such as vascular invasion or angiogenesis in patients carrying a breast cancer belonging to the HER2-enriched molecular subtype [5]. For years, the prognostic value of HER2 in the human serum (sHER2 or ECD HER2) of breast cancer patients, especially HER2 enriched cancers, has been studied [66–68]. In 2015, by monitoring sHER2 in 118 HER2 + breast cancer patients, a high sHER2 preoperative value was found to correlate with a worse prognosis [69]. Disease recurrence occurred in 12 patients over a median follow-up of 19 months. In these patients, and compared to patients who remained disease-free, higher baseline sHER2 levels were observed. In HER2 + metastatic breast cancer patients (n = 537), high serum HER2 and TIMP1 levels before any treatment predicted a short progression free survival (PFS) [70]. By evaluating serum HER2 through ELISA, Shukla et al*.* concluded that high levels were associated with poor outcomes and HER2 tissue levels in breast cancer patients [71]. The authors noted a significant correlation of high serum HER2 levels with tumour size, stage of disease and histological grade. In 2016, a retrospective study on metastatic breast cancers (HR + , HER2 + or TNBC) was conducted to investigate the predictive value of serum matrix metalloproteinases (MMP9) and HER2 [72]. HER2 and MMP-9 levels were significantly higher in the brain metastasis group (n = 88) than in the control group (n = 162). In the brain metastasis group, the overall survival (OS) was 9.8 months for patients with low serum MMP9 levels compared to 2.6 months in the high-MMP9-level patients.

### Tissue inhibitor of metalloproteinase 1

The tissue inhibitor of metalloproteinase 1 (TIMP-1) inhibits matrix metalloproteinases (MMPs) and thus may influence tumour growth and invasion. TIMP‐1 levels are higher in tumour tissues than in normal tissues and promotes cell growth, tumorigenesis, and angiogenesis [73]. The negative prognostic impact of serum TIMP-1 as well as tissue protein levels was observed in breast cancer and other cancers. Elevated serum levels of the invasion markers TIMP-1 in metastatic breast cancer are prognostic markers [74]. Müller et al*.* worked on 253 patients with metastatic breast cancer (HR + , HER2 + , and TNBC) and evaluated serum TIMP-1 concentration by means of ELISA. Median PFS was 7.2 months with high TIMP-1 and 11.4 months with low levels. It has also been shown that patients with elevated serum vascular endothelial growth factor (sVEGF) were significantly more likely to present with elevated TIMP1, and both were associated with poor OS [75]. In a 2008 study conducted on 60 breast cancer patients, high serum levels of both MMP-9 and TIMP-1 were also associated with lower progression-free and OS rates [76]. Thus, high serum levels of TIMP-1 before surgery were associated with a 53% OS rate and a 42% relapse-free survival rate. When serum TIMP-1 levels were low, the OS rate and the relapse-free survival rate were respectively 82% and 83%. More recently, TIMP-1 levels were described as prognostic factors of shorter PFS in patients with a metastatic HER2 + breast cancer treated with lapatinib or trastuzumab [70]. Serum HER2 and TIMP-1 before any treatment were quantified using ELISA in 472 patients. The median PFS in patients with high TIMP-1 levels was shorter (approximately 8 months) than in the group with low TIMP-1 levels (approximately 11 months).

### LRP6N

LRP6 ectodomain (LRP6N) functions as a co-receptor for Wnt signal induction and is involved in activating the β-catenin–dependent canonical Wnt signalling pathway, known to promote tumour progression. A team analysed LRP6N serum levels in vitro and in vivo in patients with metastatic breast cancer and demonstrated that it could be a diagnostic marker for the early detection of breast cancer metastasis [77]. Indeed, serum LRP6N was downregulated in the metastasis groups (both in mice and in patients with breast cancer). Moreover, LRP6N inhibits SDF-1/CXCR4 signal transduction involved in metastasis promotion.

### Hepatocyte growth factor

Hepatocyte growth factor (HGF) is involved in various biological processes such as morphogenesis, cell and tissue survival, and cellular growth. By activating its receptor cMet, it also facilitates tumour invasion and metastasis. In 1995, serum HGF in hormone receptor positive or negative breast cancer patients (n = 134) was studied using ELISA [78]. High levels correlated with the appearance of metastases, and 29 of 35 patients with recurrent breast cancer had an increase in serum HGF level. In a similar way, serum HGF, measured by ELISA, was correlated with a poor prognosis and a high risk of progression in metastatic (n = 34) breast cancer patients [79]. 56% (6 patients) of patients had died in the high HGF group and only 9% (2 patients) had died in the low HGF group. Later, in an attempt to demonstrate the prognostic role of HGF in breast cancer, Kim et al. used ELISA to measure the HGF serum levels of 121 female patients before neoadjuvant treatment [80]. They found that high levels of HGF correlated with metastasis. Paradoxically, they were also associated with a long relapse-free survival (106 vs. 85 months).

### Vascular endothelial-cadherin

VE-cadherin, also known as cadherin 5, plays a crucial role in endothelial adherens junction assembly and maintenance. It controls the integrity and permeability of vessels [81]. Labelle et al. demonstrated that VE-cadherin could stimulate the TGF-β pathway—involved in cell tumour proliferation—and thus promote tumour progression [82]. In primary luminal, HER2 + and TN breast cancers (n = 48), assessment of VE-cadherin serum levels is sufficient to distinguish recurrent cancers from non-recurrent ones [83]. VE-cadherin levels were elevated in sera from patients with metastatic breast cancer compared to those from patients with no recurrence. More interestingly, serum VE-cadherin appears as a prognostic factor for both PFS and OS in hormone-resistant metastatic breast cancer (n = 141) [84]. An elevated serum VE-cadherin level was associated with shorter PFS (median PFS = 9.7 months vs 5.8 months) and OS (median OS = 34 months vs 14.8 months) than was a normal or low one.

### Matrix metalloproteinase 9

Matrix metalloproteinases (MMPs) are intracellular zinc (Zn2+) dependent endopeptidases [85]. They are involved in the degradation of extracellular matrix (ECM) proteins such as collagen or fibronectin and help in the extracellular matrix remodelling in physiological and pathological processes [86]. MMP levels are increased in many cancers and are associated with increased metastases and poor clinical outcomes [87]. MMP9 expression is high in breast cancer tissues [88, 89].

The potential of MMP9 for the prediction of metastasis in BC has been explored over the past few years. Many studies demonstrated a correlation between high serum MMP9 levels and metastasis in BC patients [72, 76, 90–92]. In a study conducted in 2014, the serum levels of MMP-9 in 77 patients with breast cancer were quantified through ELISA [91]. MMP9 levels were significantly higher in the breast cancer group, regardless of BC subtype, compared to the benign tumour group (n = 10). As previously written, Darlix et al*.* demonstrated that elevated serum HER2 and MMP9 levels were associated with brain metastases in HER2 + breast cancer patients [72].

Moreover, a high tissue MMP9 level is a clear feature of TNBC and is correlated with poor prognosis [39]. Wang et al. investigated the predictive and prognostic value of MMP9 for patients with TNBC (n = 303). Using ELISA, they measured serum MMP9 levels at baseline and before surgery in patients with a non-pathological complete response [93]. High serum MMP9 correlated with a decrease in pathological complete response (pCR) rate and thus with a worse response to NAC. Interestingly, they demonstrated that each 1 ng/ml decrease in sMMP-9 after NAC was shown to result in a 0.3% increase in pCR rate. They also demonstrated that serum MMP9 levels are concordant with MMP9 protein levels detected by immunohistochemistry. However, histological MMP9 levels appeared to be a slightly better prognostic marker than serum levels.

### Vascular endothelial growth factor

Angiogenesis factors, such as VEGF, have been extensively studied in cancers. Indeed, angiogenesis influences the development and the spread of cancer. And VEGF is well known to play an important role in tumour angiogenesis, blood vessel permeability and metastases [94, 95]. In 2006, a study using ELISA demonstrated a close correlation between serum VEGF level and disease prognosis [96]. In 44 patients, the VEGF level at day one after breast surgery decreased significantly compared to its level before surgery. At day 120 after surgery, only 15 patients had higher VEGF levels than before or right after surgery. Globally, late clinical stages were found in these 15 patients with high VEGF levels [96]. Many studies emphasize that high VEGF plasma levels are associated with a poor outcome in breast cancer patients [75, 97, 98]. For instance, in a study of 253 metastatic breast cancer patients, those with elevated levels of sVEGF had significantly worse clinical outcomes [75]. Specifically, median PFS and median OS were 4.8 months and 10.2 months respectively for patients with high sVEGF levels against a PFS of 9.1 months for patients with lower sVEGF levels while median OS had not been reached in this group. In 2003, Bachelot et al. conducted a study on 87 patients with hormone-resistant metastatic breast cancer in order to investigate the prognostic value of serum and plasma VEGF. The median survival was 9 months for patients with low VEGF levels versus 13 months for patients with high VEGF levels [98].

VEGF is highly expressed in around 30–60% of patients with triple negative breast cancer, a heterogeneous cancer that accounts for 10–20% of all breast cancers. In a clinical study conducted by Taha et al. on 21 patients, serum VEGF-A levels were measured at baseline, and after the 3rd and 6th NAC cycles in patients with metastatic TNBC [99]. Serum VEGF-A at baseline—in particular, high levels thereof—was associated with a better overall survival (10.2 months versus 4.2 months in the low VEGF levels group). These results highlight the prognostic value of VEGF. Later, Bahhnassy et al. [63] quantified serum VEGF-A expression before and after standard chemotherapy using ELISA. The study was conducted on 43 TNBC patients, 53 non-TNBC patients and 20 normal control participants. A high expression of VEGF in TNBC patients correlated with a worse response to NAC, metastasis and a poorer OS (median OS around 22 months). More recently, serum levels of VEGF have been demonstrated to predict NAC response of patients with TNBC [100]. Wang et al. analysed serum samples of 303 TNBC patients prior to NAC, prior to the third cycle of NAC and prior to surgery. They found that an increase in serum VEGF prior to the third cycle of NAC has a predictive value for pCR with high sensitivity and high specificity. It is also an interesting predictor of non-response. Interestingly, high levels of VEGF are also associated with an unfavourable outcome, with a 3-year disease-free survival (DFS) of 53% (vs 85% with low serum VEGF levels).

### TIE-1/2 and angiopoietin 2

The angiopoietins family is composed of secreted proteins that all bind to the endothelial receptor called Tie2 (tyrosine kinase with immunoglobulin and epidermal growth factor homology domains 2) and are mediated by both Tie1 and Tie2. Ang-2 is notably involved in angiogenesis in presence of VEGF. And Tie2 controls tumour development (angiogenesis and growth) and metastatic production through the Ang/Tie axis [101]. Over years, the correlation between Ang-2 levels and the progression and/or outcome of patients with various cancers has been investigated [102–105]. Several studies demonstrated that aberrant expression of Ang-2 promotes tumorigenesis and cancer progression [102, 103]. A study conducted by Li et al*.* showed that serum Ang-2 in breast cancer patients (n = 143) was higher than in the healthy group (n = 100) and was associated with a worse OS and metastasis [106]. These results were similar regardless of breast cancer subtypes. In the high Ang-2 expression group, the 5-year OS and the 5-year disease-free survival rates were 55.9% and 46.0% respectively, while they were 83% and 68.7% in the low Ang-2 expression group. These results highlighted the potential of serum Ang-2 as an early detection and prognostic biomarker in patients with breast cancer. In 2011, serum angiogenesis factors were investigated to assess their diagnostic value and their association with the clinico-pathological data of 127 breast cancer patients [107]. Serum Ang-2 was clearly overexpressed in breast cancer patients compared to benign breast disease patients, but there was no evidence of an existing relationship between serum Ang-2 levels and clinical-pathologic parameters of breast cancer. Interestingly, the authors did not observe any correlation between histological type and angiogenesis markers levels. In a similar way, in metastatic breast cancer, a team analysed serum angiogenesis- and hypoxia-associated proteins to determine their potential association with patient outcomes [108]. Among other proteins, high Ang-2 levels at baseline were significantly associated with poor PFS. Moreover, both Ang-2 and serum Tie2 appeared as prognostic factors of poor OS. Tiainen et al*.* examined the concentration of Ang-2 and took interest in the soluble extracellular domain of Tie1 (sTie1) in the plasma of patients with metastatic breast cancer (n = 58) [109]. Concentrations of both sTie1 and Ang-2 were measured before, during and after the first-line treatment by ELISA assay. A high baseline Tie1 level was found to have a significant prognostic value and was associated with short median OS (around 20 months) and short median PFS (around 10 months). Moreover, patients with both high baseline Tie1 and Ang-2 levels had the worst median OS (21.5 months vs 46.8 months for patients with low Tie1 and Ang-2).

### Insulin growth factor 1 and platelet-derived growth factor

Insulin like growth factor I (IGF I) is an important regulator of growth, survival, migration and invasion and is clearly implicated in BC. Indeed, high serum levels of IGF I were associated with metastasis and recurrence in TNBC patients [52]. By analysing the serum of 110 postmenopausal breast cancer patients, Pasanisi et al. showed the impact of serum IGF I and platelet-derived growth factor (PDGF) levels on the risk of recurrence [110]. The hazard ratio (HR) of recurrence of the high PDGF group with reference to the low PDGF group was 2.8. The HR of recurrence for high IGF-I versus the low PDGF group was 3.7. Interestingly, they observed that IGF-I may increase this risk of recurrence in the presence of high serum PDGF levels. PDGF is another growth factor known to interact and act synergistically with IGF-I. Together, they promote cell proliferation [110].

### Haptoglobin

Haptoglobin (HP) is a plasma glycoprotein that binds free haemoglobin, prevents the loss of iron and inhibits oxidative activity [111]. Overexpression of serum HP has been detected in many cancers, including gastric cancer, oesophageal cancer, leukaemia, bladder cancer, and lung cancer [112]. In some studies, high serum levels of HP were also observed in patients with BC and have been linked to poor clinical outcomes [113]. A study analysing serum HP expression in patients with TNBC suggested that it could be a potential biomarker [114]. Indeed, Tabassum et al*.* correlated blood HP levels with patient outcomes by demonstrating that patients with the highest levels had the worst prognosis and the lowest survival rate. After 44 months, 54.0% of low HP-level patients had DFS. They also pointed to the possibility of using haptoglobin as a therapeutic target for TNBC. In a study published in 2014, serum proteomes were examined by mass spectrometry in TNBC patients (n = 30) and non-TNBC patients (n = 30) and a screening of differentially expressed proteins was performed [115]. In the two groups, serum samples were collected prior to any treatment. Serum haptoglobin, but also transthyretin (TTR) and antitrypsin (A1AT), had a significantly increase in expression in the TNBC group. Thus, haptoglobin, TTR and A1AT are credible candidates for the early detection of TNBC. However, the authors emphasized the need to investigate its role as a TNBC biomarker more precisely. Recently, in a human triple-negative breast cancer xenograft model, dramatic up-regulation of plasma haptoglobin was observed after metastasis, suggesting the potential of HP as a biomarker for metastasis [116]. They also noticed a significant decrease in serum HP before the development of metastases.

### Cancer antigen 15–3 and carcinoembryonic antigen

Serum cancer antigen 15–3 (CA15-3) and carcinoembryonic antigen (CEA) are prognostic markers for BC that have been studied for decades. CA15-3 is a member of the mucin-1 family of glycoproteins that are overexpressed in cancers [117]. CEA is a cell-surface glycoprotein—identified as a tumour-specific antigen—and a clinically useful tumour marker in some adenocarcinomas. It is the most widely accepted serum breast tumour marker.

In 2016, Dai et al. collected and analysed serum CA15-3, CEA levels and other factors of patients (n = 247) with TNBC prior to NAC [118]. They showed that elevated CEA (HR: 2.293) and CA15-3 (HR: 2.627) levels were associated with shorter OS than those in the low-level groups. They were also associated with short DFS rates. Similarly, a study conducted on 604 TNBC patients aimed to analyse serum CEA, CA15-3 and other factors [119]. High serum levels of these two proteins were associated with a high risk of death. The authors demonstrated that serum CEA and CA15-3 were independent prognostic factors for OS in patients with TNBC. Importantly, tumour location, number of positive lymph nodes, and histological grade also appeared as significant prognostic factors. Recently, however, a retrospective study conducted by Nam et al*.* showed discordant results [120]. They measured CA15-3 and CEA serum levels prior to surgery in BC patients. The CA15-3 and CEA-elevated group had a HR of 2.14. The CA15-3-elevated group had an HR of 2.38 and the CEA-elevated group had an HR of 1.79 compared to the normal group. However, while serum CEA and CA15-3 elevation appear to be significant prognostic factors in luminal breast cancers; this is not the case for TNBC. The authors justify the conflict of their results with those of Dai et al. by noting that the latter included stage IV patients in their study and, thus, results may be dependent on the stage of patients’ cancers.

### Cancer PD-1/PD-L1

Programmed cell death protein 1 (PD-1) is an immune checkpoint receptor that regulates T-cell activation and immune surveillance [121]. Programmed death ligand 1 (PD-L1) is the principal ligand of programmed death 1 (PD-1), a co-inhibitory receptor that can be constitutively expressed or induced in myeloid, lymphoid, and normal epithelial cells as well as in cancer. Under physiological conditions, the PD-1/PD-L1 interaction is essential in the development of immune tolerance, as it prevents excessive immune cell activity that can lead to tissue destruction and autoimmunity [121]. PD-L1 expression is an immune evasion mechanism exploited by numerous cancers and is also suggested as a predictive biomarker of response to immunotherapies. Over recent years, PD-1/PD-L1 inhibitory therapies have developed rapidly and have gained interest as novel anticancer therapeutics in different types of cancers such as metastatic lung cancer, melanoma and many others [121]. In breast cancer patients, PD-L1’s expression in cancer cells is associated with response to NAC and OS [103, 104]. Interestingly, PD-L1 in exosomes and more specifically soluble PD-L1 appears as a credible predictive and prognostic marker to monitor treatment efficacy in a myriad of cancers including breast, gastric, urothelial, esophageal or hepatocellular cancers [124–130]. In addition, serum PD-L1 mRNA expression in blood mononuclear cells could be associated with disease progression in breast cancer [131]. In TNBC, Li and his team [132] took an interest in soluble PD-L1 and PD-1. Their serum concentrations were quantified through ELISA in 66 TNBC patients treated with neo-adjuvant chemotherapy. As a result, before NAC, high levels of PD-L1 (227.7 pg/mL) and PD-1 (549.3 pg/mL) were observed in TNBC patients compared to healthy women (195.0 pg/mL and 379.2 pg/mL respectively). More interestingly, TNBC patients with a complete or partial response presented significantly lower serum PD-L1 and PD-1 levels after NAC compared to before NAC. Serum PD-1 and PD-L1 are predictive factors of treatment response in TNBC patients. A recent study evaluated the prognostic and predictive value of serum PD-L1 levels in HER2-positive metastatic breast cancer treated with trastuzumab or lapatinib [133]. High serum PD-L1 level before treatment was strongly linked to longer OS in the lapatinib group compared to the trastuzumab group. A recent study took interest in the prognostic value of sPD-L1 in patients with metastatic breast cancer [134]. sPD-L1 levels were quantified by ELISA in 208 patients. The authors found that elevated sPD-L1 level (> 8.774 ng/ml) before first-line treatment was associated with a short PFS of metastatic breast cancer and a poor prognosis [134]. However, in a recent study, Yazdanpanah et al*.* found no difference in serum levels of sPD-L1between TNBC patients (n = 72) and healthy women (n = 40) and no correlation between tumour PD-L1 expression and sPD-L1 in TNBC patients [135].

## Circulating proteins produced by the immune system

### Interleukin-6

Interleukin 6 (IL-6) is a cytokine produced in particular by endothelial cells and normal haematopoietic cells. IL-6 is involved in the upregulation of acute phase response proteins implicated in various processes including cell proliferation or inflammation. IL-6 clearly acts as a tumour promotion and progression factor. For example, IL-6 regulates the *TP53* gene and this way promotes growth [136]; it also mediates tumour invasion and metastasis and many other tumour processes. Interestingly, IL-6 could be implicated in the production of VEGF and more specifically in its upregulation. In 2003, as mentioned above, Bachelot et al. tested the prognostic value of serum levels of interleukin 6 and vascular endothelial growth factor in 87 hormone-resistant metastatic breast cancer patients [98] and identified IL6 as a potential prognostic factor of survival. High serum IL-6 levels were associated with a poor survival. Indeed, high serum IL-6 levels were associated with a median survival of 4 months, whereas patients with low serum IL-6 levels had a median survival of 13 months. A study conducted on 110 patients diagnosed with ductal carcinoma highlighted the correlation of high serum IL-6 and IL-8 levels with advanced clinical disease stages and lymph node metastasis [137]. In 2017, sera from 110 breast cancer patients were collected and evaluated for serum levels of sonic hedgehog (Shh) and IL-6 independently of their progesterone, estrogen or HER2 status [28]. Both high serum levels of Shh and IL-6 had a significantly higher risk of recurrence and metastasis, and were associated with a worse chance of survival. Median survival was significantly shorter for patients with high serum IL-6 and Shh levels (10.2 and 12 months respectively).

### Cytokines

More globally, as mentioned above, inflammation and cancer are intrinsically associated. Changes in cytokine levels influence cancer development and progression. For instance, a team determined a correlation between serum angiogenesis- and hypoxia-associated proteins and patient outcome in breast cancer patients treated with paclitaxel and bevacizumab without or with capecitabine [108]. They showed, in 181 patients, that high IL-6 and IL-8 levels at baseline were associated with poor progression-free survival and poor OS. Interestingly, patients who responded to the treatment presented a relative decrease in IL8 levels whereas a relative increase was observed in non-responders (median relative change 19.4% vs. 22.3%). Li et al. analysed serum cytokine profiles in breast cancer patients through ELISA [52]. Among other cytokines, IL-8, Macrophage Inflammatory Proteins-1 alpha and Macrophage Inflammatory Proteins-1 beta were significantly higher in breast cancer patients. These cytokines could be useful inflammatory markers in breast cancer patients. More recently, Sparano et al.demonstrated an association between serum IL-6 and an elevated risk of distant recurrence in HER2 + patients [138]. In 2022, a study conducted on 72 premenopausal ER + breast cancer patients evaluated the prognostic significance of serum IFN-γ. The authors classified the patients into two groups depending on baseline serum levels of IFN-γ: IFN-γ^high^ subgroup and IFN-γ^low^ subgroup [139]. Interestingly, distant recurrence incidence was 4% for the IFN-γ^high^ subgroup and 33% for the IFN-γ^low^ subgroup. Elevated serum IFN-γ levels were associated with favourable disease outcome in ER + BC.

### LCN2

The glycoprotein Lipocalin 2 (LCN2), also called neutrophil gelatinase-associated lipocalin, is mainly known to act as a factor limiting bacterial growth involved in innate immunity. It is notably well-expressed in neutrophils and was initially discovered in complex with matrix metalloproteinase-9 (MMP-9) [140]. We know that it participates in acute organ injury and is specifically used as an acute kidney injury marker [141]. But LCN2 appears to play a role in various biological processes such as inflammatory response, transport of some lipophilic molecules and cancer growth. Today, some studies highlight its role in the development of some cancers and especially of human breast cancer. A study led by Provatopoulou et al. focused on the circulation of LCN2 and MMP9 at diagnosis in 113 women with breast cancer [142]. Higher serum levels of LCN2 were observed in breast cancer patients compared to healthy women. In invasive ductal carcinoma, high concentrations—measured by ELISA—correlated with a high severity score. Moreover, MMP9 and LCN2 serum levels were correlated. In 2012, a Korean team also found that patients (n = 303) with elevated serum levels of LCN2 or MMP-9 at diagnosis had a poor disease-free survival [143]. High MMP9 and LCN2 serum levels were correlated and associated with poor DFS, respectively or in association.

## Conclusions

This review summarizes recent literature on plasma or serum proteins described as potential predictive and prognostic biomarkers in breast cancer. Blood protein studies in breast cancer have mostly evaluated the capacity of circulating proteins to predict patient outcome. For example, high serum levels of TIMP1 are promising indicators of poor overall survival of breast cancer patients [76], whereas high serum levels of MMP-9 have been demonstrated to strongly predict response to treatment and metastasis [72, 144]. The assessment of MMP9 and LCN2 serum levels could be of great interest to predict PFS but further investigations need to be done to ensure the clinical relevance of this association [143]. Moreover, high serum levels of VEGF and TGF-β1 remain important biomarkers of short OS, either used alone or in combination with other proteins such as Ang-2 and its mediator Tie-2 [106, 107, 109]. Serum PD-L1 appears as an interesting candidate biomarker in cancer immunotherapy, worth further validation [122, 132, 133]. Serum HER2 is also a reliable prognostic biomarker in HER2 + BC, nevertheless tissue HER2 remains more clinically relevant. Interestingly and globally, we can see similar observations across studies on the role and the clinical relevance of each of the proteins presented in our review. This overall homogeneity confirms the importance of conducting further investigations. However, BC biomarker studies are mostly based either on a single protein or on a group of proteins with similar functions. We believe that the study of a panel of proteins with various functions could strengthen the prognostic and predictive value of the biomarkers highlighted in this review. This is the objective of an ongoing study in our team which focuses on the predictive value of a group of proteins including TGF- β1, VEGF, MMP9, TIMP1 or PD-L1 (NCT04438681) [145].

The proteins found in blood have a long-standing history as cancer biomarkers, longer than several classes of biomarkers introduced more recently for liquid biopsy such as ctDNA, or miRNA. Blood profiling has recently gained momentum, especially in search for biomarkers useful in immuno-oncology and in monitoring of cancer patient response to neoadjuvant treatments [146–149]. However, despite technological improvements, protein detection sensitivity remains slightly poorer than ctDNA detection sensitivity. Consequently, liquid biopsies should explore proteins but should be complemented by other usual biomarkers such as ctDNA or circulating tumour cells [150]. Contrary to proteins biomarkers, ctDNA can also identify new mechanisms of therapy resistance and potential new targets for treatment. Thus, liquid biopsy exploring composite biomarkers could describe the overall proteomic and genomic landscape of the tumour, both in space and in time which gives it a definite advantage compared to the more invasive tissue biopsy. Moreover, it can easily be repeatedly performed during patient follow-up, and could facilitate the assessment of treatment efficacy. For now, liquid biopsy is not routinely used in clinical practice despite the growing interest in implementing it into the multidisciplinary decision-making. However, it is being increasingly evaluated in translational studies in oncology, so it is expected to improve the personalized management of cancer patients in the coming years.

## Data Availability

Not applicable.

## Abbreviations

Ang2: Angiopoietin 2
BC: Breast cancer
CA15-3: Cancer antigen 15-3
CEA: Carcinoembryonic antigen
DFS: Disease-free survival
ELISA: Enzyme-linked immunosorbent assay
HER2: Human epidermal growth factor receptor 2
HGF: Hepatocyte growth factor
HR: Hazard ratio
HP: Haptoglobin
IGF: Insulin-like growth factor
IL6/8/10: Interleukin 6/8/10
Lipocalin 2: LCN2
LRP6N: LRP6 ectodomain
MALDI-TOF: Matrix-assisted laser desorption/ionisation time-of-flight
MMPs: Matrix metalloproteinases
MS: Mass spectrometry
NAC: Neoadjuvant chemotherapy
OS: Overall survival
pCR: Pathological complete response
PD1: Programmed cell death protein 1
PDGF: Platelet-derived growth factor
PDL1: Programme death ligand 1
PFS: Progression free survival
SELDI-TOF: Surface-enhanced laser desorption/ionisation time-of-flight
TGF β: Transforming growth factor β
Tie-1/2: Tyrosine kinase with immunoglobulin and epidermal growth factor-homology domains 1/2
TIMP-1: Tissue inhibitor of metalloproteinase 1
TNBC: Triple negative breast cancer
TNF α: Tumour necrosis factor‐α
VEGF: Vascular endothelial growth factor
